# Tyrosine kinase inhibitor BIBF1120 ameliorates inflammation, angiogenesis and fibrosis in CCl_4_-induced liver fibrogenesis mouse model

**DOI:** 10.1038/srep44545

**Published:** 2017-03-14

**Authors:** Büsra Öztürk Akcora, Gert Storm, Jai Prakash, Ruchi Bansal

**Affiliations:** 1 Targeted Therapeutics, Department of Biomaterials Science and Technology, MIRA Institute for Biomedical Technology and Technical Medicine, Faculty of Science and Technology, University of Twente, Enschede, The Netherlands; 2 Department of Pharmaceutics, Utrecht Institute of Pharmaceutical Sciences, Faculty of Science, Utrecht University, Utrecht, The Netherlands

## Abstract

Hepatic fibrosis, a progressive chronic disease mainly caused by hepatitis viral infections, alcohol abuse or metabolic syndrome leading to liver dysfunction and is the growing cause of mortality worldwide. Tyrosine kinase inhibitor BIBF1120 (Nintedanib) has been evaluated in clinical trials for idiopathic pulmonary fibrosis and advanced Hepatocellular carcinoma, but has not been explored for liver fibrosis yet. In this study, we aimed to investigate the therapeutic effects and mechanism of BIBF1120 in liver fibrogenesis. The effects of BIBF1120 were evaluated in TGFβ-activated mouse 3T3 fibroblasts, LX2 cells, primary human hepatic stellate cells (HSCs) and CCl_4_-induced liver fibrogenesis mouse model. Fibroblasts-conditioned medium studies were performed to assess the paracrine effects on macrophages and endothelial cells. *In-vitro* in TGFβ-activated fibroblasts, BIBF1120 significantly inhibited expression of major fibrotic parameters, wound-healing and contractility. *In vivo* in CCl_4_-induced acute liver injury model, post-disease BIBF1120 administration significantly attenuated collagen accumulation and HSC activation. Interestingly, BIBF1120 drastically inhibited intrahepatic inflammation and angiogenesis. To further elucidate the mechanism of action, 3T3-conditioned medium studies demonstrated increased 3T3-mediated macrophage chemotaxis and endothelial cells tube formation and activation, which was significantly decreased by BIBF1120. These results suggests that BIBF1120 can be a potential therapeutic approach for the treatment of liver fibrosis.

Liver fibrosis is characterized by the extensive accumulation of abnormal extracellular matrix (ECM) proteins leading to liver dysfunction^1^,^2^,^3^. Hepatocellular damage, inflammatory cells infiltration and extensive tissue remodelling, vascular disorganisation and tissue hypoxia ultimately culminate into progressive fibrosis, cirrhosis or end-stage liver failure, leading to high morbidity and mortality worldwide^1^,^2^,^3^. Despite our increasing understanding of cellular and molecular mechanisms contributing to liver fibrosis or cirrhosis, no anti-fibrotics are currently licensed for use in humans^4^,^5^. Activated hepatic stellate cells (HSCs or liver myofibroblasts) are the main effector cells involved in the pathogenesis of liver fibrogenesis^6^. In normal liver, quiescent HSCs store vitamin A in lipid droplets, but in response to liver injury, damaged hepatocytes and inflammatory cells released growth factors and pro-fibrogenic cytokines, HSCs undergo characteristic morphological and functional changes to transform to proliferative, contractile and ECM-producing myofibroblasts characterized by the loss of vitamin droplets^6^.

Macrophages, found in close proximity to HSCs also play a key role in fibrosis initiation and progression^7^,^8^. Hepatic macrophages can arise either from circulating bone-marrow derived monocytes, which are recruited to the injured liver, or from proliferating resident macrophages (Kupffer cells). Resident macrophages have shown to play a role in initiating inflammatory responses during tissue injury, while infiltrating monocyte-derived macrophages leads to chronic liver inflammation and fibrogenesis^8^,^9^,^10^. Both resident and recruited macrophages produce pro-fibrotic mediators and chemokines that directly activate and recruit fibroblasts and inflammatory cells and control ECM turnover by regulating the balance of matrix metalloproteinases (MMPs) and tissue inhibitors of matrix metalloproteinases (TIMPs)^7^,^8^. During recruitment due to liver damage, inflammatory monocytes undergo differentiation into two distinct subsets of macrophages that are categorized as classically-activated inflammatory M1-like macrophages activated by Th1 cytokines e.g. IFN-γ, TNF-α or IL-12, or alternatively-activated resolving M2-like macrophages stimulated by Th2 cytokines IL-4 or IL-13^9^. There exists a positive interplay between HSCs and inflammatory macrophages strongly contributing to amplification of fibrosis^8^. We have also recently shown a Notch-mediated cross-talk between fibroblasts and macrophages in liver fibrosis^11^. Additionally, recent data have revealed that angiogenesis contributes significantly to the progression of fibrosis during the wound healing process in chronic liver damage^12^,^13^,^14^. A critical link between angiogenesis, inflammation and fibrosis has been investigated during chronic liver diseases^13^,^15^. HSCs activated in response to hypoxia, play a key role in angiogenesis through interactions with endothelial cells via PGDF and VEGF signaling^16^. These data suggests the multidimensional role of HSCs i.e. exaggerating inflammation and angiogenesis in liver fibrosis^6^,^16^. Liver sinusoidal endothelial cells (LSECs) has shown to play a critical role in liver fibrosis^17^,^18^. LSECs are required to maintain HSCs quiescence, but during liver fibrosis, LSECs become highly pro-inflammatory and are a critical component of intrahepatic inflammation^19^. Thus, there is an intricate interplay between the non-parenchymal cells that either prevents or sustains HSC activation and thereby determines fibrosis^17^.

HSCs are also important source of growth factors e.g. Platelet-derived growth factor (PDGF), Transforming growth factor (TGF) and epidermal growth factor (EGF) that stimulate HSCs in autocrine manner and also activate other key cell types via paracrine signaling^6^,^16^. PDGF signaling pathway is a well-known pathway for HSC activation. PDGF is the most potent mitogen for HSCs and induction of its receptor, PDGFβR, is a hallmark of early HSCs activation, followed by HSCs transformation to contractile phenotype, and correlates with the degree of fibrosis^20^. PDGF antagonism and pharmacological inhibition of PDGFβR has shown to be a promising therapeutic approach^21^,^22^. Receptors for vascular endothelial growth factors (VEGF) and fibroblast growth factor (FGF) are also shown to be induced during HSC activation that contribute to angiogenesis and fibroblasts activation respectively^16^. BIBF1120 (or Nintedanib) is a triple tyrosine kinase or tyrosine kinase inhibitor that blocks key HSCs receptors e.g. FGFR, PDGFR and VEGFR. BIBF1120 has been tested previously and showed potential therapeutic effects in a preclinical idiopathic pulmonary fibrosis (IPF) models and in clinical trials^23^,^24^. However, BIBF1120 and its mechanism of action remained unexplored in liver fibrosis.

In this study, we explored the therapeutic effects of BIBF1120 *in vitro* in 3T3 fibroblasts, LX2 cells, primary human hepatic stellate cells and *in vivo* in CCl_4_-induced acute liver fibrogenesis mouse model. Using conditioned medium studies, we further investigated the antagonistic effects of BIBF1120 on fibroblasts resulting in inhibitory paracrine effects on macrophages and endothelial cells.

## Results

### Tyrosine kinase inhibitor BIBF1120 inhibited PDGF-BB induced proliferation and TGFβ-mediated activation of mouse fibroblasts *in vitro*

**Figure 1:**
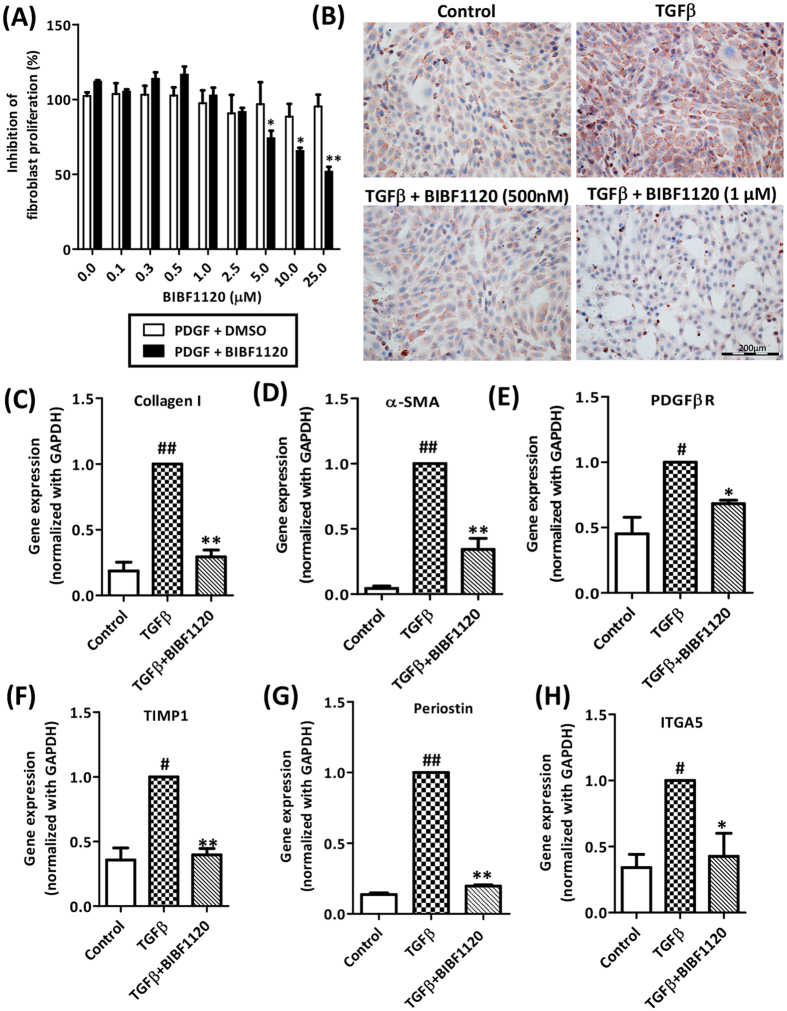
Effect of tyrosine kinase inhibitor BIBF1120 on proliferation and activation of mouse 3T3 fibroblasts *in vitro.* (**A**) Graph depicts % inhibition of fibroblasts proliferation after 24 h of treatment with DMSO (control) or BIBF1120 at different concentrations (0.1, 0.25, 0.5, 1.0, 5.0, 10.0, 25.0 μM) in combination with PDGF (50 ng/ml). n = 3. *p < 0.05, **p < 0.01 denotes significance versus control cells. (**B**) Representative images (scale bars, 200 μm) of collagen I stained 3T3 fibroblasts treated with or without TGFβ (5 ng/ml) ± 500 nM or 1 μM tyrosine kinase inhibitor (BIBF1120). Gene expression analysis (normalized with GAPDH) of fibrotic parameters on 3T3 fibroblasts treated with or without TGFβ (5 ng/ml) ± 1 μM tyrosine kinase inhibitor (BIBF1120): (**C**) collagen I, (**D**) α-SMA (alpha smooth muscle actin), (**E**) PDGFβR (Platelet-derived growth factor beta receptor), (**F**) TIMP1 (Tissue inhibitor of matrix metalloproteinases 1), (**G**) Periostin and (**H**) ITGA5 (Integrin alpha 5). Bars represent mean ± SEM, n = 3. #p < 0.05, ##p < 0.01 denotes significance versus control cells; *p < 0.05, **p < 0.01 denotes significance versus TGFβ-treated cells.

We first examined the anti-proliferative effects of tyrosine kinase inhibitor in mouse fibroblasts. We found that BIBF1120 inhibited PDGF-induced cell proliferation in 3T3 fibroblasts (Fig. 1A). We further examined the effect of BIBF1120 on TGFβ-induced collagen deposition and activation of fibroblasts. We observed that following TGFβ activation, there was a significant increase in protein and gene expression of major fibrotic parameter (collagen-I) (Fig. 1B,C). Treatment with BIBF1120 induced significant dose-dependent reduction in collagen-I protein expression in TGFβ-activated mouse 3T3 fibroblasts (Fig. 1B) and also a substantial inhibition in the respective mRNA expression levels was observed (Fig. 1C). Furthermore, BIBF1120 led to the inhibition of TGFβ-stimulated fibroblasts activation as depicted by attenuation of mRNA expression levels of α-SMA (alpha smooth muscle actin); PDGFβR (Platelet derived growth factor beta receptor) and TIMP1 (Tissue inhibitor of matrix metalloproteases 1) (Fig. 1D–F). Periostin is a TGFβ superfamily-responsive matricellular protein, produced by fibroblasts, has been shown to be important for collagen fibrillogenesis and overall organization of ECM. We also analyzed the effects of BIBF1120 on Periostin expression and observed substantial upregulation of Periostin in response to TGFβ which was drastically reduced to normal levels following incubation with BIBF1120 (Fig. 1G). We also assessed the effects on the fibronectin receptor, Integrin alpha 5 (ITGA5) since it has been shown that α5β1 is highly expressed in fibroblasts and promotes fibroblast motility and survival^25^,^26^. We found that BIBF1120 inhibited TGFβ-stimulated ITGA5 expression (Fig. 1H). No significant effect on cell viability was found at these concentrations (500 nM and 1 μM) of BIBF1120 as can also be seen in Supplementary Fig. S1A.

### Tyrosine kinase inhibitor BIBF1120 inhibited differentiation, migration and contractility of LX2 cells *in vitro*

**Figure 2:**
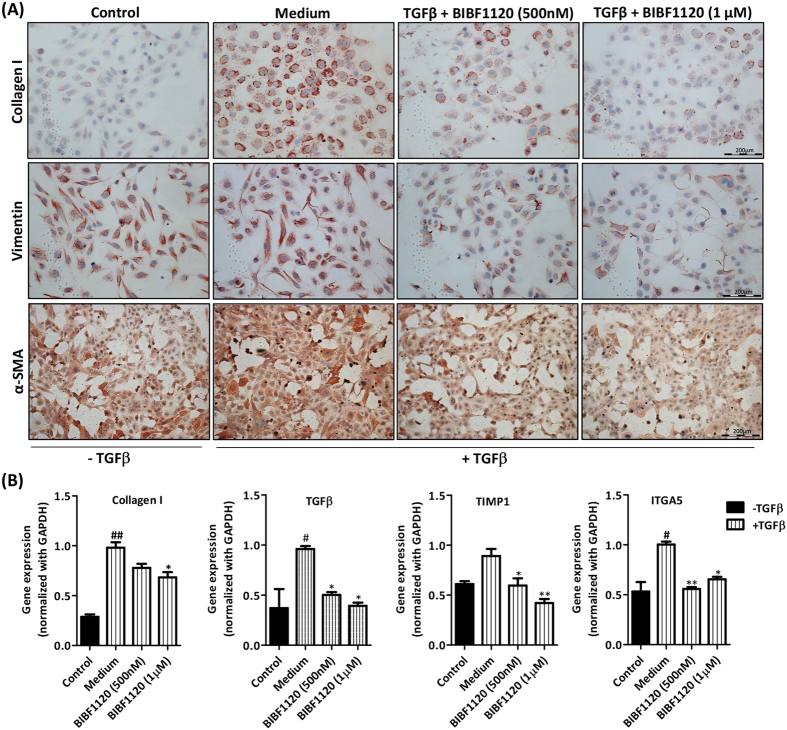
Effect of tyrosine kinase inhibitor BIBF1120 on activation of TGFβ-activated human LX2 cells. (**A**) Representative images (scale bars, 200 μm) of collagen I, Vimentin and α-SMA stained LX2 cells treated with or without TGFβ (5 ng/ml) ± 500 nM or 1 μM tyrosine kinase inhibitor (BIBF1120). (**B**) Quantitative gene expression analysis (normalized with GAPDH) of fibrotic parameters on LX2 cells treated with or without TGFβ (5 ng/ml) ± 500 nM or 1 μM tyrosine kinase inhibitor (BIBF1120): collagen I, TGFβ1 (transforming growth factor beta 1), TIMP1 (Tissue inhibitor of matrix metalloproteinases 1) and ITGA5 (Integrin alpha 5). Bars represent mean ± SEM, n = 3. #p < 0.05, ##p < 0.01 denotes significance versus control cells; *p < 0.05, **p < 0.01 denotes significance versus TGFβ-treated cells.

We further performed the studies in human hepatic stellate cell line (LX2). Following TGFβ mediated activation of LX2 cells, we observed significant upregulation of protein expression of ECM molecules i.e. collagen I and Vimentin, and HSCs activation marker i.e. α-SMA (Fig. 2A). Inhibition of tyrosine kinase signaling pathway using BIBF1120 resulted in dose-dependent inhibition in protein expression levels of these fibrogenic parameters (Fig. 2A and Supplementary Fig. S2A,B). We further examined the effect of BIBF1120 on gene expression of major fibrosis-related parameters Collagen I, TGFβ1, TIMP1 and ITGA5 and observed highly significant down-regulation in the expression levels of these parameters as shown in Fig. 2B. In addition, we examined, using western blot analysis, protein expression of Collagen I, fibronectin receptor integrin alpha 5 (ITGA5)^27^ and Integrin-signaling molecule pFAK^28^ and found that expression of these parameters were significantly reduced (Supplementary Fig. S3A–C). No significant effect on cell viability was found at these concentrations (500 nM and 1 μM) of BIBF1120 (Supplementary Fig. S4).

**Figure 3:**
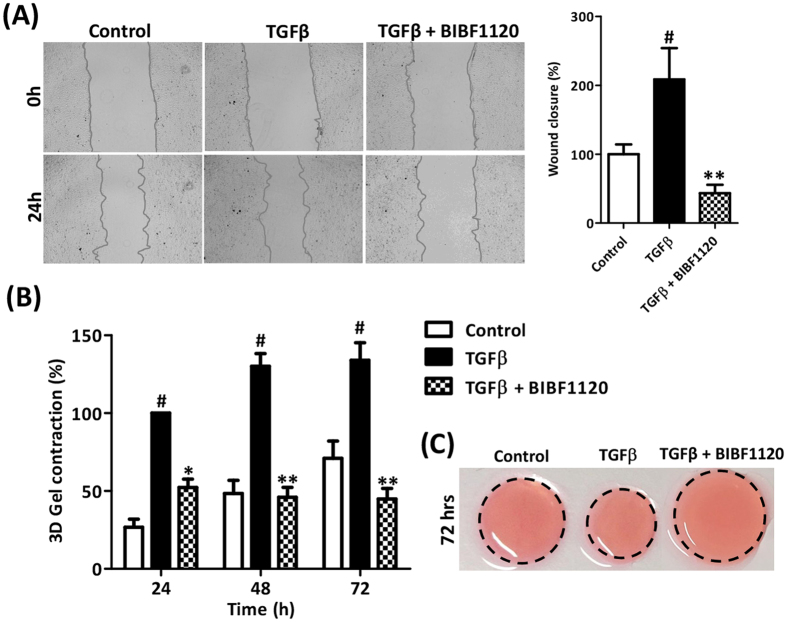
Effect of tyrosine kinase inhibitor BIBF1120 on wound healing and contractility of TGFβ-activated human LX2 cells. (**A**) Representative images and quantitative analysis (expressed as % wound closure) of scratch wounds made at 0 hr and wound closure at 24 hr as performed on control LX2, LX2 treated with TGFβ (5 ng/ml) ± 1 μM BIBF1120. Bars represent mean ± SEM, n = 3. #p < 0.05 denotes significance versus control LX2 cells. **p < 0.01 denotes significance versus TGFβ-treated LX2 cells. (**B**) Quantitative analysis (expressed as % contraction) at different time points and (**C**) representative images (after 72 h of incubation) of 3D collagen-I gel contraction containing control LX2 cells and TGFβ-activated LX2 cells treated with or without BIBF1120 (1 μM). Bars represent mean ± SEM, n = 3. #p < 0.01 denotes significance versus control LX2 cells. *p < 0.05, **p < 0.01 denotes significance versus TGFβ-treated LX2 cells.

Since HSCs can migrate to the sites of tissue injury during fibrogenesis and differentiate into contractile myofibroblasts that promote liver stiffness^29^, we further examined the effect of BIBF1120 on migration using wound-healing assays and contractility of LX2 cells using 3D-collagen contraction assay. We found that 1 μM BIBF1120 significantly inhibited TGFβ-induced migration of LX2 cells after 24 hrs as shown in Fig. 3A. Furthermore, BIBF1120 drastically diminished TGFβ-induced collagen gel contraction after 24 h, 48 h and 72 h (Fig. 3B). Maximal inhibitory effects were observed after 72 h of incubation as depicted in Fig. 3C. To confirm if these effects are not related to differences in proliferation, we performed Alamar blue assay at different times of incubations both in wound healing and gel contraction assays and did not observe any differences in proliferation following BIBF treatment.

### Tyrosine kinase inhibitor BIBF1120 inhibited differentiation of primary human hepatic stellate cells *in vitro*

**Figure 4:**
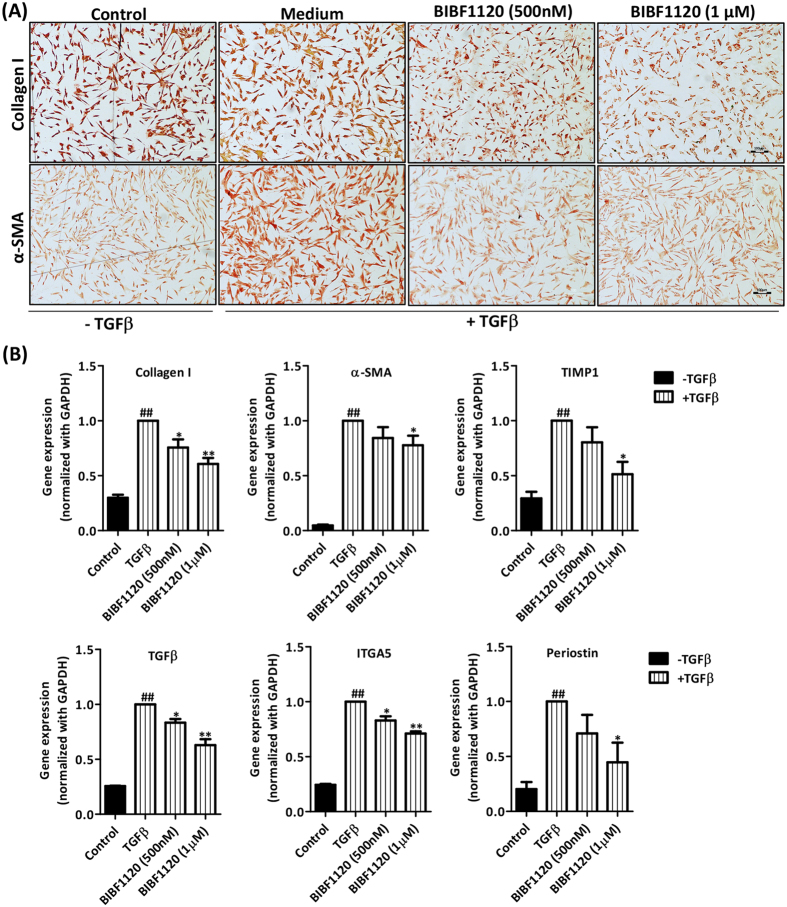
Effect of tyrosine kinase inhibitor BIBF1120 on activation of TGFβ-activated primary human hepatic stellate cells (pHSCs). (**A**) Representative images (scale bars, 100 μm) of collagen I and α-SMA stained pHSCs treated with or without TGFβ (5 ng/ml) ± 500 nM or 1 μM tyrosine kinase inhibitor (BIBF1120). (**B**) Quantitative gene expression analysis (normalized with GAPDH) of fibrotic parameters on pHSCs treated with or without TGFβ (5 ng/ml) ± 500 nM or 1 μM tyrosine kinase inhibitor (BIBF1120): collagen I, α-SMA (alpha smooth muscle actin), TIMP1 (Tissue inhibitor of matrix metalloproteinases 1), TGFβ1 (transforming growth factor beta 1), ITGA5 (Integrin alpha 5) and periostin. Bars represent mean ± SEM, n = 3. ##p < 0.01 denotes significance versus control cells; *p < 0.05, **p < 0.01 denotes significance versus TGFβ-treated pHSCs.

We further extrapolated our studies in primary human hepatic stellate cells (pHSCs) to demonstrate the role and importance of tyrosine kinase signaling pathway during human liver fibrosis. We therefore further investigated the effect of BIBF1120 on the activation of human pHSCs. Following TGFβ mediated activation, we observed significant upregulation of protein expression of collagen I and α-SMA (Fig. 4A). Inhibition of tyrosine kinase signaling pathway using BIBF1120 resulted in significant inhibition in collagen I and α-SMA protein expression (Fig. 4A and Supplementary Fig. S5A,B). We further examined the effect of BIBF1120 on gene expression of major fibrosis-related parameters in pHSCs i.e. Collagen I, α-SMA, TIMP1, TGFβ1, ITGA5 and Periostin, and observed highly significant down-regulation in the mRNA expression levels of these parameters (Fig. 4B).

### Tyrosine kinase inhibitor BIBF1120 inhibited ECM accumulation and HSC activation in acute liver injury mouse model

**Figure 5:**
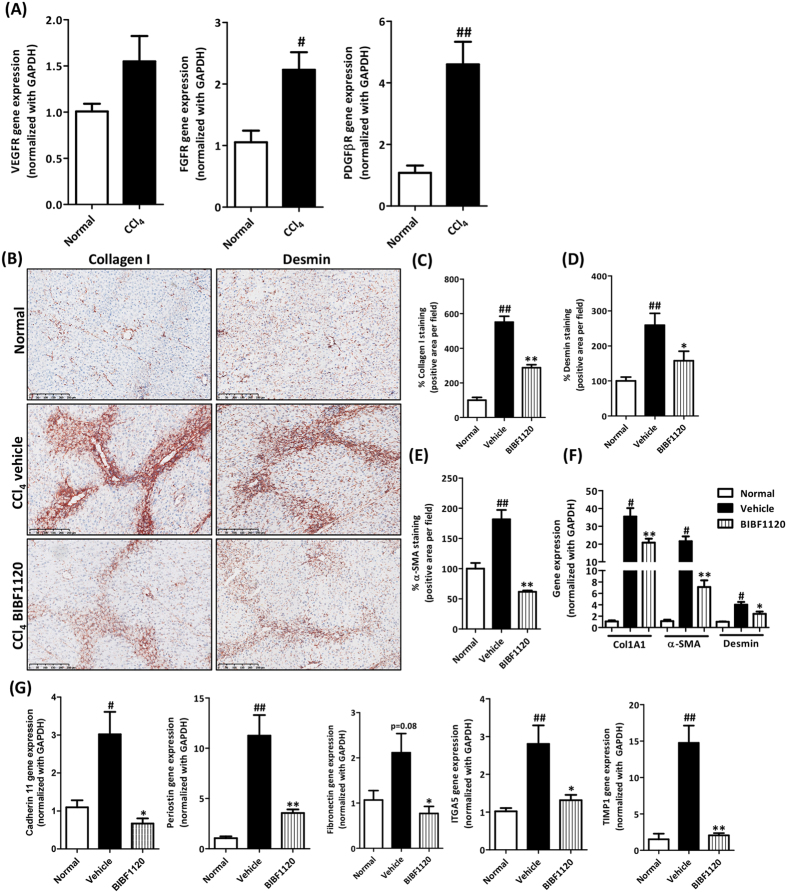
Effect of BIBF1120 inhibitor on CCl_4_-induced acute liver fibrogenesis. (**A**) Quantitative gene expression analysis of VEGFR, FGFR and PDGFβR in CCl_4_-induced acute liver fibrogenesis mouse model as compared to normal healthy controls. (**B**) Representative photomicrographs (Scale bar, 250 μm) and (**C**) Quantitative histological analysis of (**C**) Collagen I, (**D**) Desmin and (**E**) α-SMA stained liver sections from olive-oil-treated controls (normal), vehicle-treated CCl_4_ and BIBF1120-treated CCl_4_ mice. (**F**) Quantitative gene expression (normalized with GAPDH) of fibrotic parameters (Col1α1, α-SMA and Desmin) and (**G**) Cadherin 11, Periostin, fibronectin, ITGA5 (Integrin alpha 5) and TIMP1 (Tissue inhibitors of matrix metalloproteinases 1) in the livers of olive-oil-treated controls (normal), vehicle-treated CCl_4_ mice and BIBF1120-treated CCl_4_ mice. Bars represent mean ± SEM of n = 5 mice per group. #p < 0.05, ##p < 0.01 denotes significance versus olive-oil treated control group; *p < 0.05, **p < 0.01 denotes significance versus CCl_4_-treated vehicle group.

We further investigated the anti-fibrotic effects of BIBF1120 on early fibrogenesis in CCl_4_-induced acute liver injury mouse model. We first examined the expression of different receptors which are regulated by tyrosine kinase inhibitor BIBF1120 in CCl_4_-induced acute liver injury mouse model. We found that VEGFR (vascular endothelial growth factor receptor); FGFR (fibroblasts growth factor receptor) and PDGFβR (platelet derived growth factor receptor beta) were upregulated following fibrosis induction (Fig. 5A). We then investigated the effects of BIBF1120 on Collagen I expression and HSCs activation *in vivo.* Post-disease intra-peritoneal treatment with BIBF1120 (5 mg/kg) induced significant down-regulation of early fibrogenesis as shown by the significant decrease in protein and gene expression of major extracellular matrix (ECM) component, collagen I and HSCs activation markers (desmin and α-SMA) in BIBF1120 treated mice as compared to untreated CCl_4_ mice (Fig. 5B–F). Alanine aminotransferase (ALT) levels were also measured and we observed highly significant increase in ALT levels following single administration of CCl_4_ while no significant differences were observed in alanine aminotransferase (ALT) levels following treatment with BIBF1120 (Supplementary Fig. S6).

Furthermore, we investigated the effects of BIBF1120 on other crucial ECM proteins e.g. Cadherin 11^30^, fibronectin, Periostin, ITGA5 and observed strong inhibition in the expression of these parameters following BIBF1120 treatment as depicted in Fig. 5G. Furthermore, ECM regulatory protein, tissue inhibitor of metalloproteinases 1 (TIMP1) was highly reduced following BIBF1120 treatment (Fig. 5G). Altogether, these results suggests that inhibition using BIBF1120 results in the highly significant downregulation of ECM accumulation, HSCs activation and amelioration of liver fibrogenesis.

### BIBF1120 inhibited angiogenesis in acute liver injury mouse model

**Figure 6:**
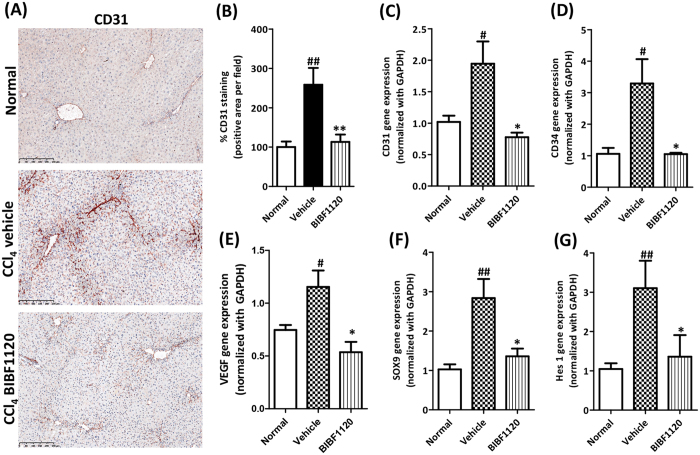
Effect of BIBF1120 on angiogenesis in acute liver injury mouse model. (**A**) Representative photomicrographs (Scale bar, 250 μm) and (**B**) Quantitative histological analysis of CD31 stained liver sections from olive-oil-treated controls (normal), vehicle-treated CCl_4_ and BIBF1120-treated CCl_4_ mice. Quantitative gene expression (normalized with GAPDH) of angiogenesis markers CD31 (**C**), CD34 (**D**) and VEGF (**E**), SOX9 (**F**) and Hes 1 (**G**) expression in the livers of olive-oil-treated controls (normal), vehicle-treated CCl_4_ mice and BIBF1120-treated CCl_4_ mice. Bars represent mean ± SEM of n = 5 mice per group. #p < 0.05, ##p < 0.01 denotes significance versus olive-oil treated control group; *p < 0.05, **p < 0.01 denotes significance versus CCl_4_-treated vehicle group.

Since hepatic angiogenesis positively correlates with fibrosis i.e. angiogenesis induced by hypoxia within an injured liver appears to aggravate hepatic fibrogenesis and vice versa i.e. hepatic stellate cells produce growth factors that stimulate angiogenesis, we further investigated the effects of BIBF1120 on angiogenesis in acute liver injury mouse model. As shown in Fig. 6A–D, BIBF1120 treatment significantly inhibited fibrosis-induced expression of major pan-endothelial cell markers CD31 and CD34. Expression of VEGF (vascular endothelial growth factor) was also found to be reduced following treatment with BIBF1120 (Fig. 6E). These anti-angiogenic effects could be partially mediated via inhibition of Notch signaling pathway (Hes1, Notch downstream pathway signaling molecule) and SOX9 (sex determining region Y)-HMG box 9 (Fig. 6F,G).

### *In vivo* effects of BIBF1120 in intrahepatic inflammation and macrophages

**Figure 7:**
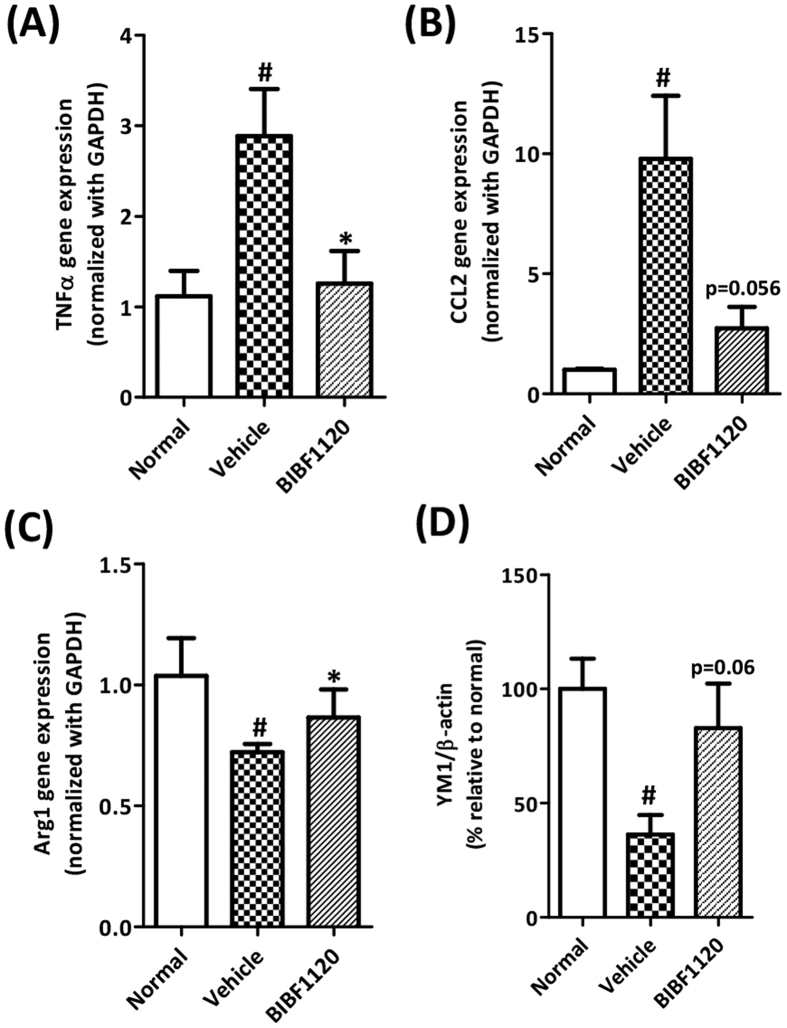
Effect of BIBF1120 on intrahepatic inflammation and macrophage polarization in CCl_4_-induced early liver fibrogenesis. Quantitative gene expression (normalized with GAPDH) of inflammation markers: (**A**) TNFα (Tumor necrosis factor alpha), (**B**) CCL2 (C-C motif chemokine ligand 2) or MCP1 (macrophage chemotactic protein 1) and (**C**) M2 macrophage marker Arg1 (arginase 1) in the livers of olive-oil-treated controls (normal), vehicle-treated CCl_4_ mice and BIBF1120-treated CCl_4_ mice. (**D**) Quantitative band intensity analysis (normalized with respective β-actin bands and expressed in %) depicting YM1 and β-actin protein expression in olive-oil-treated controls (normal), vehicle-treated CCl_4_ mice and BIBF1120-treated CCl_4_ mice. Bars represent mean ± SEM of n = 5 mice per group. #p < 0.05, denotes significance versus olive-oil treated control group; *p < 0.05 denotes significance versus CCl_4_-treated vehicle group.

As described earlier that macrophages play very crucial role in liver inflammation and development of fibrosis, we further examined the effect of BIBF1120 on liver inflammation. We analyzed gene expression of major inflammatory cytokines i.e. TNFα (tumor necrosis factor alpha) and CCL2 (C-C motif chemokine ligand 2) or MCP1 (macrophage chemotactic protein), and IL-6 (Interleukin-6) and found that intrahepatic expression of these cytokines were strongly attenuated following BIBF1120 administration (Fig. 7A,B and Supplementary Fig. S7).

Thereafter, we investigated the status of M1 (inflammatory) and M2 (restorative) macrophages in the control and BIBF1120 treated livers. As shown in Supplementary Fig. S8A–C, we found that BIBF1120 induced no change in M1 macrophages as confirmed with NOS2 (iNOS or nitric oxide synthase, specific M1 marker) gene expression and MHC-II protein expression as confirmed by western blot. Further, we analyzed expression of specific M1 markers i.e. LY-6C^31^ and IRF5^32^ using immunostainings but observed no differences (data not shown). However, BIBF1120 induced M2 macrophage polarization, as confirmed by Arg I (Arginase I, specific M2 marker) gene expression (Fig. 7C) and YM1 expression (M2 marker) (Fig. 7D, Supplementary Fig. S9).

### BIBF1120 inhibited fibroblasts-driven angiogenesis, macrophage migration and inflammation *in vitro*

As mentioned, fibroblasts (or HSCs) plays multifactorial role in liver fibrosis via different paracrine signaling pathways by release of growth factors, thereby significantly induces angiogenesis and inflammation^6^,^16^,^29^. Additionally, the positive feedback loop between fibroblasts activation, inflammation and angiogenesis further aggravate fibrosis^2^,^16^. Here, we hypothesized that BIBF1120 inhibitory effects on 3T3 fibroblasts induces paracrine inhibitory effects on angiogenesis and macrophage migration and polarization. To effectuate this, we studied the paracrine effect of BIBF1120-treated 3T3 fibroblasts on endothelial cells and macrophages. Based on previous *in vitro* and *in vivo* results, we hypothesized that secretory factors derived from fibroblasts induces angiogenesis and macrophage activation and migration and inhibition of fibroblasts using BIBF1120 inhibits secretion of these factors and therefore inhibits angiogenesis and inflammation i.e. macrophage activation/migration.

**Figure 8:**
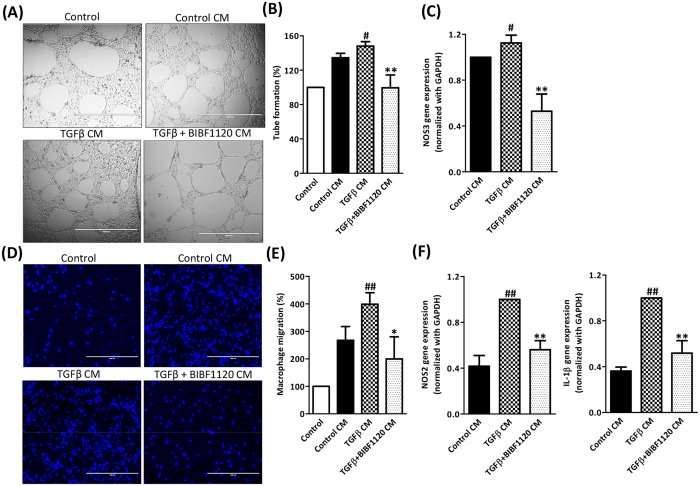
Effect of BIBF1120 on fibroblasts-driven angiogenesis (tube formation assay), macrophage migration and inflammation *in vitro.* (**A**) Representative images (Scale bar, 1000 μm) and (**B**) Quantitative analysis (expressed in %) of endothelial cell (H5V) tube formation after incubation with non-cell treated control medium (control), conditioned medium from 3T3 cells that are treated with medium alone (control CM), TGFβ (5 ng/ml) with or without 1 μM BIBF1120. (**C**) Quantitative gene expression (normalized with GAPDH) of NOS3 (nitric oxide synthase 3) in endothelial cells incubated with conditioned medium from 3T3 cells that are treated with medium alone (control CM), TGFβ (5 ng/ml) with or without 1 μM BIBF1120. (**D**) Representative images (scale bars, 200 μm) and (**E**) Quantitative analysis (expressed as % macrophage migration) of DAPI-stained migrated mouse RAW macrophages (seeded on the upper chamber on the transwell inserts) in response to non-cell treated control medium (control), conditioned medium from 3T3 cells that are treated with medium alone (control CM), TGFβ (5 ng/ml) with or without 1 μM BIBF1120 in the lower chamber. (**F**) Quantitative gene expression (normalized with GAPDH) of M1 macrophage marker, iNOS or NOS2 (nitric oxide synthase 2) and inflammation marker IL-1β in macrophages incubated with conditioned medium from 3T3 cells that are treated with medium alone (control CM), TGFβ (5 ng/ml) with or without 1 μM BIBF1120. Bars represent mean ± SEM of n = 3 independent experiments. #p < 0.05, ##p < 0.01 denotes significance versus control CM; *p < 0.05, **p < 0.01 denotes significance versus TGFβ CM.

To model fibroblasts-induced angiogenesis, we activated 3T3 fibroblasts with TGFβ with or without 1 μM BIBF1120. Thereafter, we washed them to remove the initial stimuli and then cultured in a fresh medium. After 24 hr, this conditioned medium was added to H5V endothelial cells and tube formation assay was performed. Conditioned media derived from control fibroblasts and TGFβ-stimulated fibroblasts enhanced tube formation compared to that of control (non-cell treated) medium. Interestingly, medium derived from 3T3 fibroblasts after treatment with BIBF1120 significantly inhibited the TGFβ-induced tube formation suggesting deactivation of fibroblasts using BIBF1120 inhibit fibroblasts-induced angiogenesis (Fig. 8A,B). We further analyzed the NOS3 (eNOS or nitric oxide synthase 3, endothelial marker) expression following incubation with conditioned medium and observed that conditioned medium derived from TGFβ-treated 3T3 fibroblasts induced significant upregulation in NOS3 expression which was strongly inhibited following incubation with conditioned medium derived from BIBF1120-treated 3T3 fibroblasts (Fig. 8C).

We further developed *in vitro* model to study fibroblasts-induced macrophage migration and activation. We added the conditioned medium from control 3T3 cells, TGFβ-treated 3T3 or TGFβ + BIBF1120 treated 3T3 in the lower chamber and macrophages in the upper chamber/insert to study the effect on macrophage migration through the membrane. We observed that conditioned medium from control 3T3 fibroblasts induced macrophage migration as compared to control (non-cell treated) medium suggesting chemotactic factors secreted by 3T3 fibroblasts (Fig. 8D,E). This macrophage migration was significantly enhanced in response to conditioned medium derived from TGFβ-treated 3T3 fibroblasts and was strongly inhibited following incubation with conditioned medium derived from BIBF1120-treated 3T3 fibroblasts (Fig. 8D,E). We further analyzed the expression levels of NOS2 (iNOS or nitric oxide synthase 2, M1 inflammatory macrophage marker) and IL-1β (inflammation marker). We observed that conditioned medium derived from TGFβ-treated 3T3 fibroblasts induced significant upregulation in NOS2 and IL-1β expression as compared to conditioned medium derived from 3T3 fibroblasts. This increase in expression levels were strongly inhibited following incubation with conditioned medium derived from TGFβ + BIBF1120 treated 3T3 fibroblasts (Fig. 8F).

### BIBF1120 inhibited primary human HSCs-driven LSECs and kupffer cells activation *in vitro*

We further extrapolated the conditioned medium studies using primary human hepatic stellate cells, hepatic sinusoidal endothelial cells and hepatic kupffer cells to reinforce the findings observed with 3T3 conditioned medium. We incubated hepatic kupffer cells with conditioned medium obtained from pHSCs incubated with medium, TGFβ and TGFβ + BIBF1120. We observed that TGFβ-incubated pHSCs conditioned medium significantly upregulated NOS2 and TNFα expression in primary human kupffer cells suggesting activation of primary human liver-derived macrophages (Supplementary Fig. S10A,B). This activation was abrogated following incubation with conditioned medium derived from BIBF1120-treated pHSCs (Supplementary Fig. S10A,B). Since it has been shown that LSECs are required to maintain HSCs quiescence and during liver fibrosis, LSECs secrete fibronectin, and become highly pro-inflammatory and are a critical component of intrahepatic inflammation^6^,^7^,^33^, we analyzed HSCs-mediated effect on primary LSECs. We observed that following incubation with conditioned medium derived from TGFβ-activated pHSCs led to increased expression of TNFα (inflammation marker) and fibronectin (Supplementary Fig. S11A,B). This increase was inhibited by incubation with conditioned medium obtained from BIBF1120-treated pHSCs suggesting that the soluble factors produced by pHSCs stimulate both kupffer cells and LSECs and these factors or effects of pHSCs-derived factors were inhibited by BIBF1120.

## Discussion

In this study, we investigated the therapeutic efficacy of tyrosine kinase inhibitor BIBF1120 (Nintedanib) in liver fibrogenesis. We demonstrate that tyrosine kinase inhibitor BIBF1120 strongly attenuated TGFβ-activated fibroblasts and HSC activation, collagen deposition, HSC contractility and migration *in vitro. In vivo* in CCl_4_-induced early liver fibrogenesis in mice, BIBF1120 significantly inhibited collagen deposition and HSC differentiation. Remarkably, BIBF1120 suppressed intrahepatic inflammation and angiogenesis. To understand the role of BIBF1120 in inflammation and angiogenesis, we performed conditioned medium *in vitro* studies and showed that BIBF1120 inhibited fibroblasts induced paracrine effects on macrophages (inflammation) and endothelial cells (angiogenesis). This preliminary pre-clinical investigative study exploring BIBF1120 in liver fibrosis suggests the potent inhibitory effects of BIBF1120 and suggests that BIBF1120 should be explored in the clinical trials for the treatment of liver fibrosis.

Liver fibrogenesis is a chronic progressive disease, characterized by scar formation due to the increased production and deposition of ECM proteins^1^,^2^,^16^,^34^. The key fibrogenic effector cell type is the activated hepatic stellate cells also known as myofibroblasts. HSCs have matrix producing functions and contractile properties resulting in liver distortion and dysfunction. HSCs also make a major contribution to the important aspects of the wound healing process including regeneration, inflammation and angiogenesis^6^,^29^,^34^. Upon activation due to liver damage, activated HSCs secrete growth factors e.g. PDGF, FGF, TGF, EGF, VEGF and chemokines i.e. MCP1, IL-6 etc. These mediators stimulates HSCs in autocrine fashion and also stimulates other cell types i.e. hepatocytes, macrophages and endothelial cells in paracrine manner important for liver regeneration, intrahepatic inflammation and angiogenesis respectively. HSCs are also shown to induce the expression of several receptors importantly PDGFRα and PDGFRβ, VEGFR and FGFR and are important for the autocrine activation^16^,^34^. Simultaneous blocking of these receptors will therefore induce significant de-differentiation of HSCs and therefore would influence downstream processes like intrahepatic inflammation and angiogenesis.

BIBF1120 (or Nintedanib) is a potent tyrosine inhibitor that blocks PDGFR, VEGFR and FGFR. It has been shown in lung fibroblasts that apart from these receptors, BIBF1120 has inhibitory effects on Lck, Lyn as analyzed using *in vitro* kinase assays^24^. BIBF1120 has been extensively examined in lung fibrosis mouse models, and idiopathic pulmonary fibrosis (IPF) patients and demonstrated inhibitory effects^23^,^24^ and therefore currently actively investigated in ongoing clinical trials for IPF patients. But this potent multikinase inhibitor has not been explored in liver fibrosis. In lung fibroblasts, it has been shown that BIBF1120 exert inhibitory effects by inhibiting PDGFR phosphorylation, fibroblasts proliferation and activation^23^,^24^. In this study, we showed that BIBF1120 at increasing doses inhibit PDGF-induced fibroblasts proliferation. BIBF1120 exerted significant reduction in Collagen I expression and deposition, fibroblasts activation and matrix remodeling proteins i.e. TIMP1 (tissue inhibitors of metalloproteinases 1) inducing collagen degradation by MMPs (matrix metalloproteinases), Integrins (ITGA5) and periostin (ligand for integrins) that support cell adhesion and migration via signaling through FAK-mediated signaling pathway^28^. We further examined the specific effects of BIBF1120 in human HSCs, and found that BIBF1120 attenuated TGFβ-stimulated HSCs activation, collagen I expression, HSCs migration and contractility.

BIBF1120 has shown to exhibit anti-fibrotic and anti-inflammatory effects in lung fibrosis mouse models. In this study, we first demonstrate upregulation of PDGFR, VEGFR and FGFR in acute liver fibrogenesis mouse model. Thereafter, we analyzed BIBF1120 in acute liver fibrogenesis mouse model. Interestingly, we observed anti-fibrotic, anti-inflammatory and anti-angiogenic effects of BIBF1120. We found that BIBF1120 significantly inhibited adhesion proteins (Periostin, fibronectin, ITGA5 and cadherin-11) that are important in cell adhesion, motility and contractility which are important factors for portal hypertension since increased portal hypertension further deteriorates the liver function. Furthermore, we observed strong inhibition of angiogenesis partially exhibited due to the drastic inhibition of Notch signaling pathway and SOX9 expression that has shown to regulate many developmental processes including angiogenesis^11^,^33^,^35^. To delineate the anti-inflammatory effects, we analyzed specific M1 and M2 macrophages markers and we found that inflammatory M1 macrophages remained unchanged while there was significant upregulation in suppressive M2 macrophages suggesting BIBF1120 via indirect pathways stimulate M2-directed macrophage polarization. Since inflammatory cytokines TNFα and CCL2 play an important role in development of fibrosis^10^, the inhibition of these pro-fibrogenic factors resulted in reduced fibrosis.

To further understand the effects of BIBF1120 on angiogenesis and intrahepatic inflammation, we performed paracrine cross-talk studies with fibroblasts (and HSCs), macrophages (and kupffer cells) and endothelial cells (and LSECs). We observed that fibroblasts conditioned medium contains factors that stimulate macrophage activation, and endothelial cells activation in paracrine fashion. Interestingly, BIBF1120 incubated fibroblasts inhibited release of these paracrine factors and therefore exhibited attenuated macrophage activation, and endothelial cells activation.

However, there are number of limitations within this study. This study has been performed in acute (or mild) liver fibrogenesis model that does not correspond to the clinical situation. Patients normally presents to the clinic when liver damage progresses to cirrhosis associated with portal hypertension and liver dysfunction. Nevertheless, this study provided first proof-of-concept results suggesting BIBF1120 can be an effective therapeutic drug to be explored further in liver fibrosis. Multi-kinase inhibitors however have shown to be highly toxic since they inhibit number of crucial pathways that are involved in number of cellular processes and long-term treatment or multi-dose treatment might be needed to overcome poor pharmacokinetics of these small molecule inhibitors might lead to toxicity. Therefore, HSC-selective delivery of these inhibitors using nanocarriers should be investigated as future promising therapies for the treatment of liver fibrosis^36^,^37^.

In conclusion, we demonstrated the anti-fibrotic, anti-inflammatory and anti-angiogenic effects of BIBF1120 *in vitro* and *in vivo* in acute liver fibrogenesis mouse model. These findings unveil the novel pathways regulated by BIBF1120 in fibrosis and further studies are warranted to investigate therapeutic efficacy of BIBF1120 in advanced models of liver fibrosis and finally poses an interesting drug to be explored in the clinical trials for the treatment of liver fibrosis.

## Materials and Methods

### Cell Lines

Human hepatic stellate cells (LX2 cells), immortalized human derived cell line were kindly provided by Prof. Scott Friedman (Mount Sinai Hospital, New York). Murine H5V heart capillary endothelial cells were kindly provided by Dr. A. Vecchi (Mario Negri, Institute for Pharmacological Research, Milan, Italy). Murine NIH3T3 fibroblasts and murine RAW264.7 macrophages were obtained from the American Type Culture Collection (ATCC, Manassas, VA, USA). LX2 cells were cultured in DMEM-Glutamax (Invitrogen, Carlsbad, CA) supplemented with 10% FBS and antibiotics (50 U/ml Penicillin and 50 μg/ml streptomycin, Sigma). RAW macrophages were cultured in Roswell Park Memorial Institute (RPMI) 1640 medium (Lonza, Verviers, Belgium), and 3T3 and H5V cells were cultured in Dulbecco’s modified Eagle’s (DMEM) medium (Lonza) supplemented with 2 mM L-glutamine (Sigma, St. Louis, MO), 10% fetal bovine serum (FBS, Lonza) and antibiotics (50 U/ml Penicillin and 50 μg/ml streptomycin, Sigma).

### Primary human cells

Primary human hepatic stellate cells (pHSCs), primary human liver sinusoidal endothelial cells (pLSECs) and primary human kupffer cells were obtained from Sciencell (Sciencell, CA). pHSCs were cultured on poly(L-lysine) coated plates in stellate cell medium (SteCM, Sciencell) supplemented with stellate cells growth supplements (SteCGS), 2% FBS and antibiotics (Penicillin and streptomycin). pLSECs were cultured on fibronectin-coated plates in endothelial cell medium (ECM, Sciencell) supplemented with endothelial cells growth supplements (ECGS), 5% FBS and antibiotics (Penicillin and streptomycin). Primary human kupffer cells were cultured on poly(L-lysine) coated plates in macrophage medium (MM, Sciencell) supplemented with macrophage growth supplements (MaGS), 5% FBS and antibiotics (Penicillin and streptomycin).

### Effects of Tyrosine kinase inhibitor BIBF1120 in mouse fibroblasts

Cells were seeded in 24-well plates (3 × 10^4^ cells/well for stainings) and 12-well plates (8 × 10^4^ cells/well for quantitative PCR analysis) and cultured overnight. To assess the effects on fibrotic parameters, cells were starved for 24 h and then incubated with starvation medium alone, 1 μM BIBF1120 and 5 ng/ml of human recombinant TGFβ1 (Roche, Mannheim, Germany) for 24 h. Cells (24 well plates) were then fixed with chilled acetone:methanol (1:1) for 20 min, dried and stained for collagen-I (Supplementary Table 1). In addition, cells (12 well plates) were lysed with RNA lysis buffer to perform quantitative real-time PCR analyses. Staining’s and quantitative PCR analysis was performed at least in three independent experiments.

To assess the effects on proliferation, cells plated in 96 well plates were serum-starved for 24 h and incubated with different concentrations of BIBF1120 (01, 0.5, 1, 2.5, 5, 10 and 25 μM) with 50 ng/ml human recombinant PDGF (Peprotech) for 24 h. Control cells were treated with 50 ng/ml PDGF and DMSO. Subsequently cells were incubated with Alamar Blue reagent (Invitrogen) for 4 hrs. The results are represented as % inhibition of fibroblasts proliferation. All measurements were performed in duplicates in three independent experiments.

### Effects of Tyrosine kinase inhibitor BIBF1120 in LX2 cells and primary human HSCs (pHSCs)

Cells were seeded in 24-well plates (3 × 10^4^ cells/well for stainings) and 12-well plates (8 × 10^4^cells/well for quantitative PCR analysis) and cultured overnight. To assess the effects on fibrotic parameters, cells were starved for 24 h and then incubated with starvation medium alone, 500 nM or 1 μM BIBF1120 and 5 ng/ml of human recombinant TGFβ1 (Roche, Mannheim, Germany) for 24 h. Cells (24 well plates) were then fixed with chilled acetone:methanol (1:1) for 20 min, dried and stained for collagen-I, α-SMA or vimentin (antibodies summarized in Supplementary Table 1). In addition, cells (12 well plates) were lysed with RNA lysis buffer to perform quantitative real-time PCR analyses. Staining’s and quantitative PCR analysis was performed at least in three independent experiments.

### Scratch wound healing assay

Cells were plated in 12-well culture plates (1 × 10^5^ cells/well) for 24 h and starved overnight in 0.5% FBS containing medium. A standardized scratch was made using a 200 μl pipette tip fixed in a holder. Then, cells were washed and incubated with 1 ml of 0.5% FBS containing medium with or without TGFβ (5 ng/ml) together with 1 μM BIBF1120. To measure the migratory response of the cells into the scrape wounds, microscopic photographs were taken at 0 h and 24 h. Images were analyzed by NIH ImageJ software (NIH, Bethesda, MD) to calculate the area of scratch wound and represented as % of wound healed relative to the control wells.

### 3D collagen-I gel contraction assay

A collagen suspension (5 ml) containing 3.0 ml Collagen G1 (5 mg/ml, Matrix biosciences, Morlenbach, Germany), 0.5 ml 10x M199 medium (Sigma), 85 μl 1N NaOH (Sigma) and sterile water was mixed with 1.0 ml (2 × 10^6^) cells. Collagen gel-cells suspension (0.6 ml/well) was plated in a 24‐well culture plate and allowed to polymerize for 1 h at 37 °C. For the effect studies: polymerized gel was incubated with 1 ml of 0.5% FBS containing medium with or without TGFβ (5 ng/ml) together with 1 μM BIBF1120 followed by detachment of the gels from the culture wells. For other experiments, medium with or without TGFβ was added before gel detachment. Photographs were made with a digital camera at different time points (0, 24, 48 and 72 h). The size of the gels were digitally measured and normalized with their respective well size in each image. Gel contraction experiments were performed in duplicates in three independent experiments.

### Conditioned medium studies

3T3 fibroblasts and primary human HSCs (pHSCs) were plated in complete medium in 12-well plates and cultured overnight. Then cells were serum-starved for 24 h. Then 3T3 fibroblasts and pHSCs were incubated with or without TGFβ (5 ng/ml), with and without 1 μM BIBF1120. After 24 h, incubated cells were washed and fresh medium (0% FBS) was added. After 24 h, conditioned medium was collected and stored at −70 °C until use. 3T3-conditioned medium (from different conditions) was added on unstimulated RAW macrophages and H5V endothelial cells with an equal volume of fresh respective medium to avoid nutrient depletion effects. pHSCs-conditioned medium (from different conditions) was added on unstimulated primary human kupffer cells and pLSECs with equal volume of respective medium. After 24 h incubation with the conditioned medium, cells were lysed for quantitative PCR analysis. All the conditioned medium studies were performed in three independent experiments.

### Tube Formation Assay

The fibroblasts-mediated paracrine effects of BIBF1120 on endothelial cells (H5V) were examined using the tube formation assay. Geltrex^TM^ reduced growth factor basement membrane matrix (Invitrogen) was thawed at 4 °C overnight before use. Geltrex^TM^ matrix was added to wells of a 8-chambered slides (Lab-Tek^TM^) (150 μl/well) and then incubated at 37 °C for 1 hr to allow polymerization. H5V cells (40,000 cells/well) were seeded onto the layer of Geltrex^TM^ matrix containing the conditioned medium collected from 3T3 cells after different treatment as mentioned above. After 20 h at 37 °C, 5 random selected fields of view were captured using EVOS fluorescent microscope (AMG, Life Technologies). Tube formation was quantified by measuring the number of tubes counted and represented as relative percentage of tube formation.

### Transwell Migration Assay

The transwell migration assay was performed on RAW macrophages using a polycarbonate membrane inserts (8 μm pore size; Transwell Coster Corning Inc.). The lower chambers were filled with 650 ul medium (455 ul of conditioned medium + 175 ul of fresh medium). The conditioned medium was collected from 3T3 cells after different treatment as mentioned above. Cells (1 × 10^5^ cells/100 μl) were added in the upper chamber (or insert), then incubated at 37 °C for 24 h to allow cell migration through the membrane. After incubation, the chambers were removed and washed twice with PBS and the non-invading cells were discarded using a cotton swab. The cells were fixed in 4% paraformaldehyde, permeabilized in 100% methanol and stained using DAPI nuclear stain. 5 random selected fields of view were captured using EVOS fluorescent microscope (AMG, Life Technologies) and the number of invading cells were quantified using NIH ImageJ software and represented as relative percentage of cell migration.

### Animal Experiments

All the animal experiments in this study were performed in strict accordance with the guidelines and regulations for the Care and use of Laboratory Animals, Utrecht University, The Netherlands. The protocols were approved by the Institutional Animal Ethics Committee of the University of Twente, The Netherlands. Male 6- to 8-week old C57BL/6 mice were purchased from Harlan (Zeist, Netherlands) and kept at 12 h light/12 h dark cycles with ad libitum normal diet.

### CCl_4_-induced acute liver injury mouse modelh

To study the effect of BIBF1120, male C57BL/6 mice were treated with a single intraperitoneal injection of olive-oil or CCl_4_ (1 ml/kg in olive-oil) at day 1. At day 2 and day 3, CCl_4_-treated mice received intra-peritoneal administration of 5 mg/kg tyrosine kinase inhibitor BIBF1120 (Selleckchem, Boston, NY) prepared in 1% DMSO (Sigma) and 5% β-hydroxycyclodextrin (Sigma) or vehicle treatment (1%DMSO/5%β-hydroxycyclodextrin/PBS) (n = 5 per group). At day 4, all mice were sacrificed and livers were harvested for the subsequent analysis. Alanine Transaminase (ALT) levels in the plasma was measured using ALT Colorimetric Activity Assay Kit (Cayman Chemical Company, Michigan, MI, USA) as per manufacturer’s instructions.

### Immunohistochemistry

Liver tissues were harvested and transferred to Tissue-Tek OCT embedding medium (Sakura Finetek, Torrance, CA), and snap-frozen in 2-methyl butane chilled in a dry ice. Cryosections (4 μm) were cut using a Leica CM 3050 cryostat (Leica Microsystems, Nussloch, Germany). The sections were air-dried and fixed with acetone for 10 min. Cells or tissue sections were rehydrated with PBS and incubated with the primary antibody (refer to Supplementary Table 1) for 1 h at room temperature. Cells or sections were then incubated with horseradish peroxidase (HRP)-conjugated secondary antibody for 1 h at room temperature. Then incubated with HRP-conjugated tertiary antibody for 1 hr at room temperature. Thereafter, peroxidase activity was developed using AEC (3-amino-9-ethyl carbazole) substrate kit (Life Technologies, Carlsbad, CA) for 20 min and nuclei were counterstained with hematoxylin (Fluka Chemie, Buchs, Switzerland). For tissue sections, endogenous peroxidase activity was blocked by 3% H_2_O_2_ prepared in methanol. Cells or sections were mounted with Aquatex mounting medium (Merck, Darmstadt, Germany). The staining was visualized and the images were captured using light microscopy (Nikon eclipse E600 microscope, Nikon, Tokyo, Japan). Furthermore, sections were scanned using Hamamatsu NanoZoomer Digital slide scanner 2.0HT (Hamamatsu Photonics, Bridgewater, NJ) for quantitative histological analysis.

### Quantitative histological analysis

For quantitation, stained sections were scanned at high resolution using Hamamatsu NanoZoomer Digital slide scanner 2.0HT (Hamamatsu Photonics). High resolution scans were viewed using NanoZoomer Digital Pathology (NDP2.0) viewer software (Hamamatsu Photonics). About 20 images (100x) of each entire section (from NDP) were imported into NIH ImageJ software (NIH, Bethesda, MD) and were analyzed quantitatively at a fixed threshold.

### Western blot analysis

Cells or liver tissues were homogenized in cold RIPA buffer [50 mM Tris–HCl, 150 mM NaCl, 0.1% SDS, 0.1% Igepal in 0.5% sodium deoxycholate with 1 tablet of protease inhibitor cocktail and 1 tablet of phosphatase inhibitor (Roche Diagnostics, Mannheim, Germany) in 10 ml] on ice with a tissue homogenizer and the lysates were centrifuged at 12,000 rpm for 1 h at 4 °C. The supernatants were stored at −70 °C until use. The samples were boiled in standard protein sample buffer (Life Technologies) and subjected to SDS-PAGE with 10% Tris-glycine gels (Life Technologies) followed by protein transfer onto PVDF membrane. The membranes were developed according to the standard protocols using primary and secondary antibodies as mentioned in Supplementary Table 1. The bands were visualized using ECL detection reagent (Perkin Elmer Inc., Waltham, MA) and photographed using FluorChem M Imaging System (ProteinSimple, Alpha Innotech, San Leandro CA). Intensity of individual bands was quantified using ImageJ densitometry software, and expressed in % relative to β-actin.

### RNA extraction, reverse transcription, quantitative real time PCR and RT2 profiler PCR array

Total RNA from cells and liver tissues was isolated using GenElute Total RNA Miniprep Kit (Sigma) and SV total RNA isolation system (Promega Corporation, WI) respectively according to manufacturer’s instructions. The RNA concentration was quantitated by a UV spectrophotometer (NanoDrop Technologies, Wilmington, DE). Total RNA (1 μg) was reverse-transcribed using iScript cDNA Synthesis Kit (Bio-Rad, Hercules, CA). All the primers were purchased from Sigma-Genosys (Haverhill, UK). Real-time PCR was performed using 2x SensiMix SYBR and Fluorescein Kit (Bioline, QT615-05, Luckenwalde, Germany), 20 ng cDNA and pre-tested gene-specific primer sets (listed in Supplementary Tables 2 and 3). The cycling conditions for the BioRad CFX384 Real-Time PCR detection system were 95 °C for 10 min, 40 cycles of 95 °C/15 sec, 58 °C/15 sec and 72 °C/15 sec. Finally, cycle threshold (Ct) values were normalized to reference gene GAPDH and fold changes in expression were calculated using the 2^−ΔΔCt^ method.

### Statistical analyses

All the data are presented as mean ± standard error of the mean (SEM). The graphs and statistical analyses were performed using GraphPad Prism version 5.02 (GraphPad Prism Software, Inc., La Jolla, CA). Comparison to control group were analyzed using unpaired students’ t test while multiple comparisons between different groups were performed by one-way analysis of variance (ANOVA) with Bonferroni post-hoc test. The differences were considered significant at p < 0.05.

## Additional Information

**How to cite this article:** Öztürk Akcora, B. *et al*. Tyrosine kinase inhibitor BIBF1120 ameliorates inflammation, angiogenesis and fibrosis in CCl_4_-induced liver fibrogenesis mouse model. *Sci. Rep.* **7**, 44545; doi: 10.1038/srep44545 (2017).

**Publisher's note:** Springer Nature remains neutral with regard to jurisdictional claims in published maps and institutional affiliations.

## Supplementary Material

Supplementary Data
