# Supplementary material for: Tyrosine kinase inhibitor BIBF1120 ameliorates inflammation, angiogenesis and fibrosis in CCl4-induced liver fibrogenesis mouse model
Source: Sci Rep. 2017 Mar 14;7:44545. doi: 10.1038/srep44545 (PMC5349608; doi:10.1038/srep44545)
Supplement: Supplementary Data [file srep44545-s1.pdf]

Tyrosine kinase inhibitor BIBF1120 ameliorates inflammation, angiogenesis and fibrosis in CCl_4-induced liver fibrogenesis mouse model

Büsra Öztürk Akcora[1], Gert Storm[1,2], Jai Prakash[1], Ruchi Bansal[1*]

[1]Targeted Therapeutics, Department of Biomaterials Science and Technology, MIRA Institute for Biomedical Technology and Technical Medicine, Faculty of Science and Technology, University of Twente, Enschede, The Netherlands. [2]Department of Pharmaceutics, Utrecht Institute of Pharmaceutical Sciences, Faculty of Science, Utrecht University, Utrecht, The Netherlands.

Supplementary Table 1: Antibodies used for the immunohistochemistry

**Primary Antibody**	**Source**
Monoclonal mouse anti-α-SMA	Sigma
Polyclonal goat anti-collagen I	Southern Biotech
Monoclonal rat anti-mouse CD31	Southern Biotech
Polyclonal goat anti-desmin	Santa Cruz
Polyclonal goat anti-vimentin (sc-7557)	Santa Cruz
Monoclonal rat anti-MHC Class II: sc-59318	Santa Cruz
Polyclonal goat anti-YM-1 (Biotinylated Anti-mouse chitinase-3-like 3/ECF-L)	R and D systems
Monoclonal mouse anti-β-actin	Sigma
Polyclonal rabbit anti-ITGA5	Sigma
Polyclonal rabbit anti-phospho-FAK antibody	Cell signaling
**Secondary Antibody**	**Source**
Polyclonal goat anti-rabbit IgG	DAKO
Polyclonal rabbit anti-mouse IgG	DAKO
Polyclonal goat anti-mouse IgG	DAKO
Polyclonal rabbit anti-goat IgG	DAKO
Polyclonal goat anti-rat IgG	Southern Biotech

Supplementary Table 2: Sequence of the mouse primers used for quantitative real-time PCR

Gene	Forward primer sequence (5’-3’)	Reverse primer sequence (5’-3’)
Collagen 1α1	TGACTGGAAGAGCGGAGAGT	ATCCATCGGTCATGCTCTCT
Desmin	ATGCAGCCACTCTAGCTCGT	CTCATACTGAGCCCGGATGT
ACTA2 (α-SMA)	ACTACTGCCGAGCGTGAGAT	CCAATGAAAGATGGCTGGAA
GAPDH	ACAGTCCATGCCATCACTGC	GATCCACGACGGACACATTG
TIMP1	ATCAGTGCCTGCAGCTTCTT	TGACGGCTCTGGTAGTCCTC
PDGFβR	GCTGGAGCTGAGTGAGAGTC	GCAGGTAGACCAGGTGACAT
FGFR1	GCTGACTCCAGTGCATCCAT	ACACGGTTGGGTTTGTCCTT
VEGFR2	TAACCTGGCTGACCCGATTC	AAGTCACAGAGGCGGTATGC
CD31	TCCCTGGGAGGTCGTCCAT	GAACAAGGCAGCGGGGTTTA
CD34	GGGTAGCTCTCTGCCTGATG	TCTCTGAGATGGCTGGTGTG
Cadherin 11	CCGACTTGTGAATGGGACTCG	AGGGCCACAAAGCACAGTAA
SOX9	GTGCAAGCTGGCAAAGTTGA	TGCTCAGTTCACCGATGTCC
ITGA5	GAACCCTGTGTCCTGCATCA	TTGGAGTTCCACCTCGAAGC
Fibronectin	ATGAGAAGCCTGGATCCCCT	GGAAGGGTAACCAGTTGGGG
IL-6	TGATGCTGGTGACAACCACGGC	TAAGCCTCCGACTTGTGAAGTGGTA
TNFα	AGGCTGCCCCGACTACGTGC	CAGCGCTGAGTTGGTCCCCC
CCL2 or MCP1	GTGCTGACCCCAAGAAGGAA	GTGCTGAAGACCTTAGGGCA
TIMP1	ATCAGTGCCTGCAGCTTCTT	TGACGGCTCTGGTAGTCCTC
Arg1	GTGAAGAACCCACGGTCTGT	CTGGTTGTCAGGGGAGTGTT
NOS2 or iNOS	AATCTTGGAGCGAGTTGTGG	CAGGAAGTAGGTGAGGGCTTG
Periostin	ATCCACGGAGAGCCAGTCAT	TGTTTCTCCACCTCCTGTGG
NOS3 or eNOS	CATGGGCAACTTGAAGAGTGT	GGGTGTCGTAGGTGATGCTG
IL-1β	GCCAAGACAGGTCGCTCAGGG	CCCCCACACGTTGACAGCTAGG

Supplementary Table 3: Sequence of the human primers used for quantitative real-time PCR

Gene	Forward primer sequence (5'-3')	Reverse primer sequence (5'-3')
Collagen 1α1	GTACTGGATTGACCCCAACC	CGCCATACTCGAACTGGAAT
GAPDH	TCCAAAATCAAGTGGGGCGA	TGATGACCCTTTTGGCTCCC
PDGFβR	CATGGGGGTATGGTTTTGTC	GTAAGGTGCCAACCTGCAAT
TGFβ1	GCGTGCTAATGGTGGAAACC	GAGCAACACGGGTTCAGGTA
TIMP1	GGGGACACCAGAAGTCAACC	GGGTGTAGACGAACCGGATG
ITGA5	CAACTTCTCCTTGGACCCCC	GTCCTCTATCCGGCTCTTGC
Fibronectin	GTATACGAGGGCCAGCTCAT	CCCAGGAGACCACAAAGCTA
NOS2	CGCAGAGAACTCAGCCTCAT	TGCCTTGAGAACTTCGGGAC
TNFα	CTTCTGCCTGCTGCACTTTG	GTCACTCGGGGTTCGAGAAG
Periostin	ACAAGAAGAGGTCACCAAGGTC	CTTGCAACTTCCTCACGGGT
α-SMA	CCCCATCTATGAGGGCTATG	CAGTGGCCATCTCATTTTCA

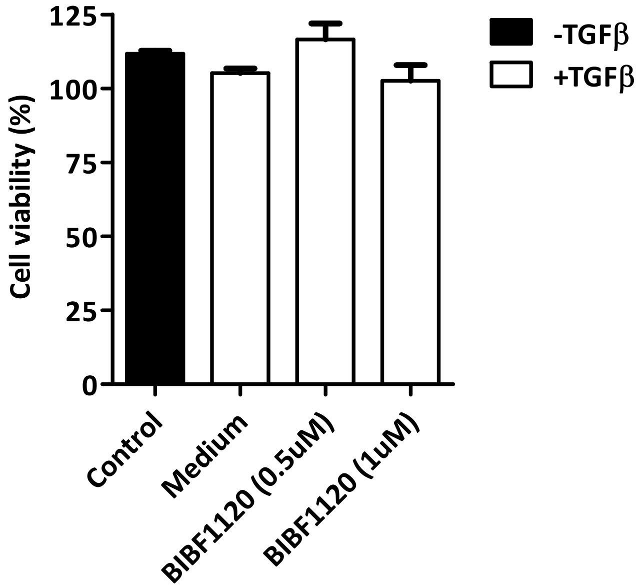

Supplementary Figure S1: Graph depicts % cell viability (as assessed by Alamar blue assay) in 3T3 fibroblasts treated with or without TGFβ (5ng/ml) ± 500nM or 1μM tyrosine kinase inhibitor BIBF1120. Bars represent mean ± SEM, n=3.

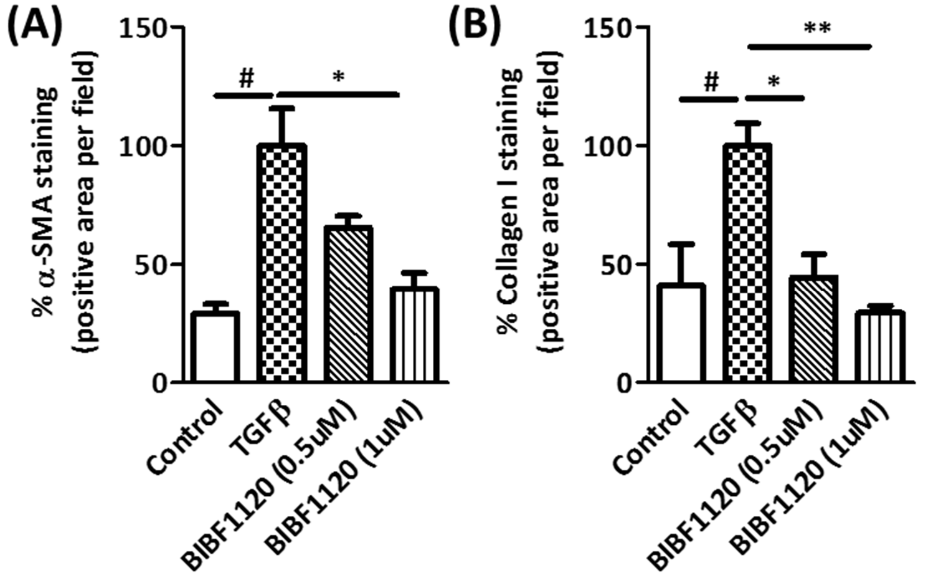

Supplementary Figure S2: Graph depicts quantitative immunostaining analysis of α-SMA **(A)** and Collagen I **(B)** stained LX2 cells that were treated with or without TGFβ (5ng/ml) ± 500nM or 1µM tyrosine kinase inhibitor BIBF1120. Bars represent mean ± SEM, n=3. #p<0.05 denotes significance versus control LX2 cells. *p<0.05, **p<0.01 denotes significance versus TGFβ-treated LX2 cells.

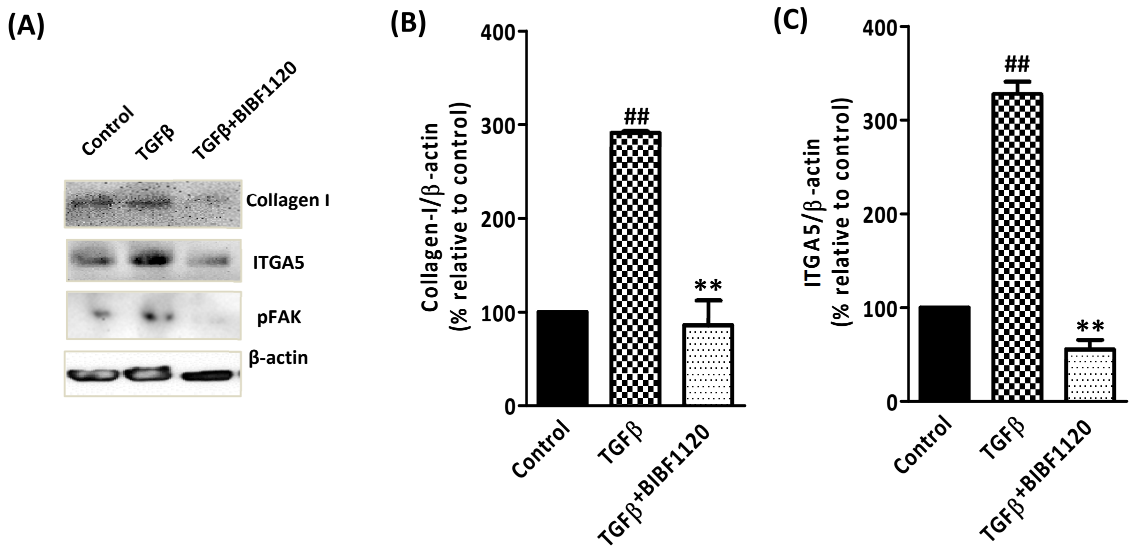

Supplementary Figure S3: **(A)** Respective western blot and **(B, C)** quantitative band intensity analysis (normalized with respective β-actin bands and expressed in %) depicting Collagen-I, ITGA5, pFAK and β-actin protein expression in human HSCs (LX2 cells) treated with or without TGFβ (5ng/ml) ± 1µM tyrosine kinase inhibitor BIBF1120. Bars represent mean ± SEM, n=3. ##p<0.01 denotes significance versus control cells; **p<0.01 denotes significance versus TGFβ-treated cells.

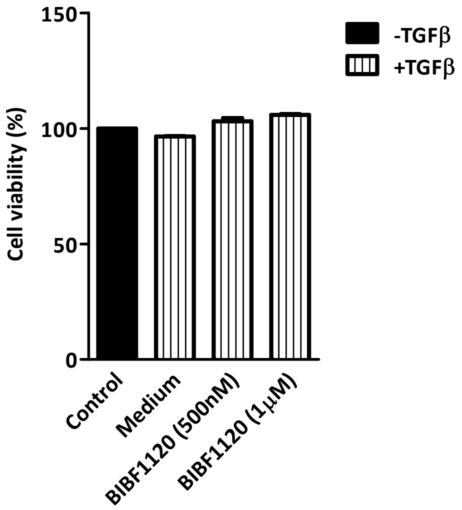

Supplementary Figure S4: Graph depicts % cell viability (as assessed by Alamar blue assay) in human LX2 cells treated with or without TGFβ (5ng/ml) ± 500nM or 1μM tyrosine kinase inhibitor BIBF1120. Bars represent mean ± SEM, n=3.

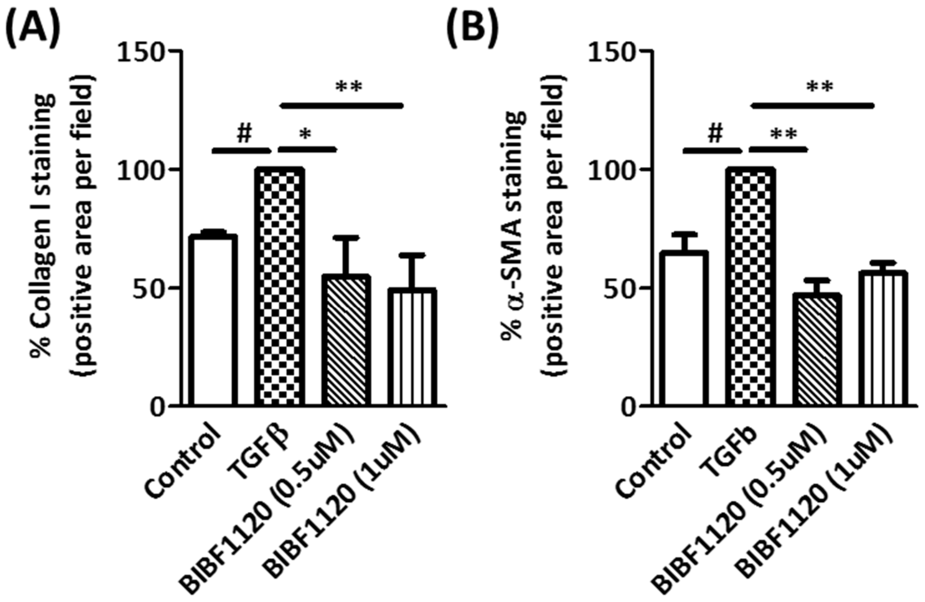

Supplementary Figure S5: Graph depicts quantitative immunostaining analysis of Collagen I and α-SMA stained primary human hepatic stellate cells (pHSCs) that were treated with or without TGFβ (5ng/ml) ± 500nM or 1μM tyrosine kinase inhibitor BIBF1120. Bars represent mean ± SEM, n=3. #p<0.05 denotes significance versus control pHSCs. *p<0.05, **p<0.01 denotes significance versus TGFβ-treated pHSCs.

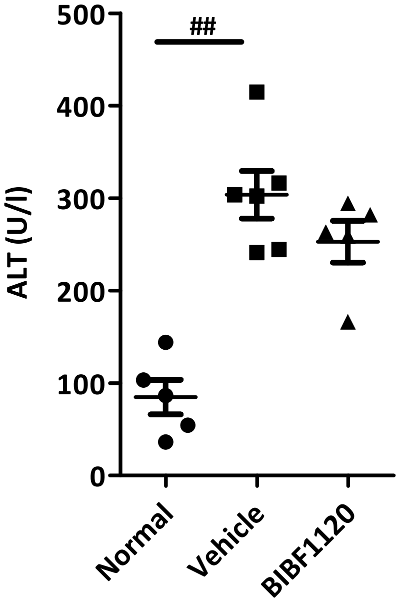

Supplementary Figure S6: Alanine aminotransferases (ALT) levels (expressed as U/l) as analyzed in the serum from olive-oil-treated controls (normal), vehicle-treated CCl_4 mice (Vehicle) and BIBF1120-treated CCl_4 mice. Each symbol represents individual mice. Bars represent mean ± SEM of n=5 mice per group. ##p<0.01 denotes significance versus olive-oil treated control group.

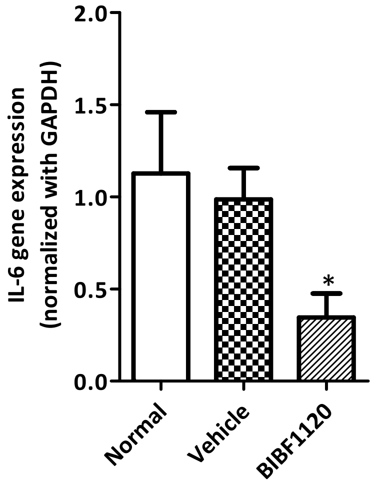

Supplementary Figure S7: Quantitative gene expression (normalized with GAPDH) of IL-6 in the livers of olive-oil-treated controls (normal), vehicle-treated CCl_4 mice and BIBF1120-treated CCl_4 mice. Bars represent mean ± SEM of n=5 mice per group. *$p<0.05$ denotes significance versus CCl_4-treated vehicle group.

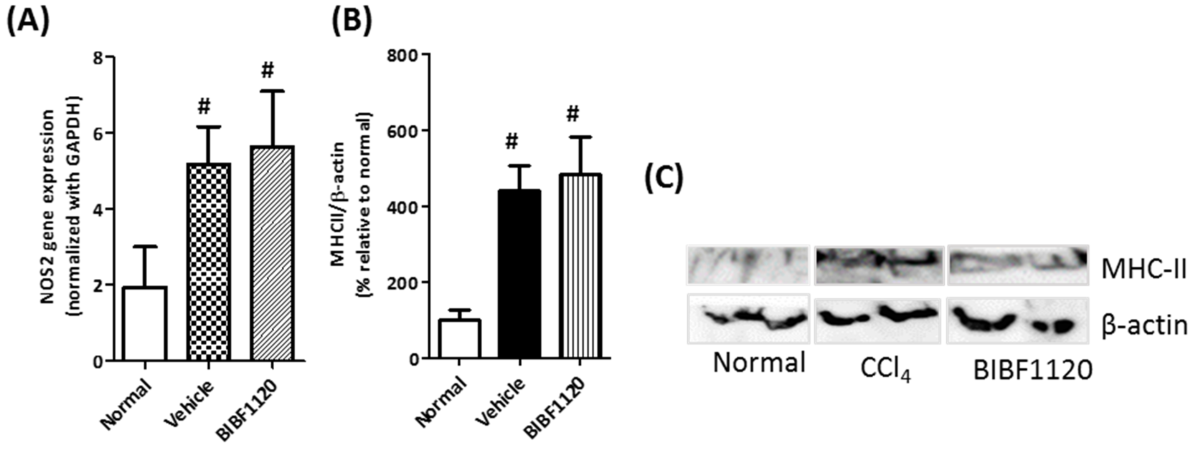

Supplementary Figure S8: **(A)** Quantitative gene expression (normalized with GAPDH) of NOS2 or iNOS in the livers of olive-oil-treated controls (normal), vehicle-treated CCl_4 mice and BIBF1120-treated CCl_4 mice. **(B)** Quantitative band intensity analysis (normalized with respective β-actin bands and expressed in %) and **(C)** Respective western blot depicting MHC-II and β-actin protein expression in olive-oil-treated controls (normal), vehicle-treated CCl_4 mice and BIBF1120-treated CCl_4 mice. Bars represent mean ± SEM of n=5 mice per group. #p<0.05, ##p<0.01 denotes significance versus olive-oil treated control group.

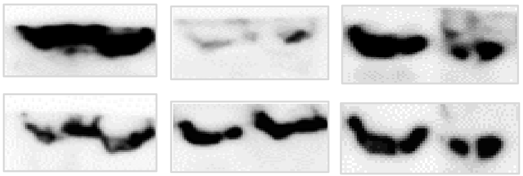

Supplementary Figure S9: Respective western blot depicting YM1 and β-actin protein expression in olive-oil-treated controls (normal), vehicle-treated CCl_4 mice and BIBF1120-treated CCl_4 mice.

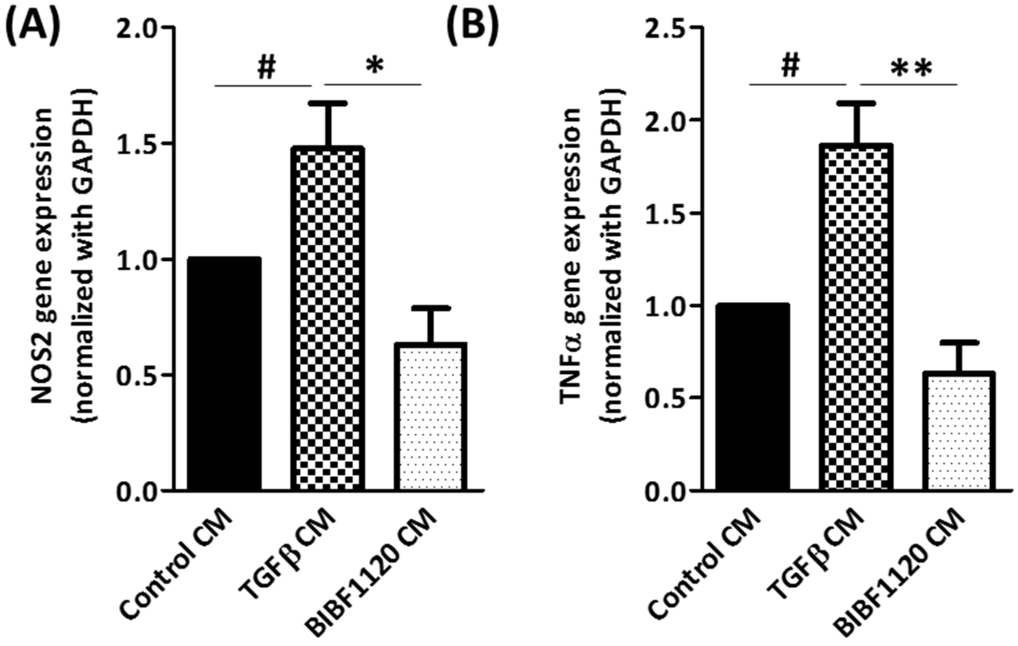

Supplementary Figure S10. Quantitative gene expression (normalized with GAPDH) of M1 macrophage marker, iNOS or NOS2 (nitric oxide synthase 2) **(A)** and inflammation marker TNFα **(B)** analyzed in primary human kupffer cells incubated with conditioned medium from primary human hepatic stellate cells that are treated with medium alone (control CM), TGFβ (5ng/ml) with or without 1µM BIBF1120. Bars represent mean ± SEM of n=3 independent experiments. #$p<0.05$ denotes significance versus control CM; *$p<0.05$, **$p<0.01$ denotes significance versus TGFβ CM.

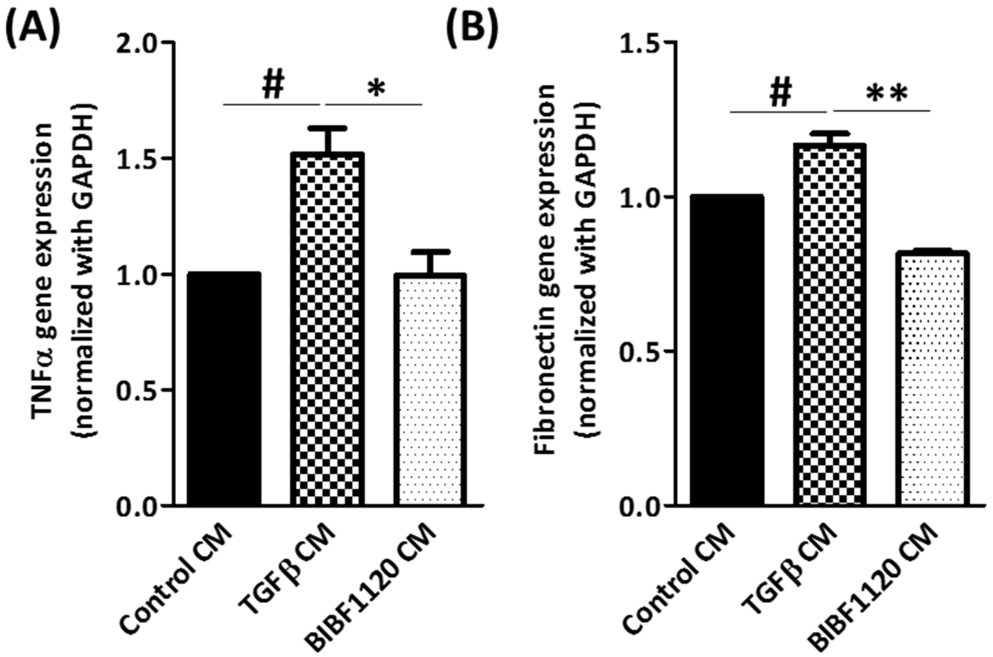

Supplementary Figure S11. Quantitative gene expression (normalized with GAPDH) of inflammation marker TNFα **(A)** and fibronectin **(B)** analyzed in primary human LSECs incubated with conditioned medium from primary human hepatic stellate cells that are treated with medium alone (control CM), TGFβ (5ng/ml) with or without 1µM BIBF1120. Bars represent mean ± SEM of n=3 independent experiments. #$p<0.05$ denotes significance versus control CM; *$p<0.05$, **$p<0.01$ denotes significance versus TGFβ CM.
